# Neutron crystallography aids in drug design

**DOI:** 10.1107/S2052252516013439

**Published:** 2016-08-31

**Authors:** M. P. Blakeley

**Affiliations:** a Large-Scale Structures Group, Institut Laue-Langevin, 71 Avenue des Martyrs, Grenoble, 38000, France

**Keywords:** human carbonic anhydrase, acetazolamide, methazolamide, neutron crystallography, drug design

## Abstract

Since drugs bind to their targets through directional H bonding and non-directional hydrophobic and electrostatic interactions, neutron crystallography can help guide structure-based drug design. This is illustrated by McKenna and co-workers [Aggarwal *et al.* (2016), *IUCrJ*, **3**, 319–325] who describe the room-temperature neutron structure of human carbonic anyhydrase II in complex with the clinical inhibitor methazolamide to 2.2 Å resolution, and compare this with the previously determined room-temperature neutron structure of human carbonic anyhydrase II in complex with the clinical inhibitor acetazolamide to 2.0 Å resolution [Fisher *et al.* (2012). *J. Am. Chem. Soc.***134**, 14726–14729].

Neutron crystallography is an important complementary technique to X-ray crystallography since it provides details of the hydrogen (H) atom and proton (H^+^) positions in biological molecules. Furthermore, as neutrons (of the energies used for crystallographic experiments) are a non-destructive probe, the resulting structures are free from radiation damage even at room temperature (Blakeley *et al.*, 2015 ▸). Knowledge of H-bonding networks, water molecule orientations and protonation states, along with details of hydro­phobic and electrostatic interactions, can prove vital towards a better understanding of many biological processes, such as enzyme mechanisms (Casadei *et al.*, 2014 ▸; Vandavasi *et al.*, 2016 ▸) and ligand binding (Howard *et al.*, 2016 ▸), and can help guide structure-based drug design (Huang *et al.*, 2014 ▸).

The first neutron crystallography study of a clinically used drug bound to its target was that of acetazolamide (AZM), a sulfonamide, which binds with high affinity to human carbonic anhydrase isoform II (Fisher *et al.*, 2012 ▸). Human carbonic anhydrases (hCA) are zinc metalloenzymes that catalyze the interconversion of CO_2_ and H_2_O to HCO_3_^−^ and H^+^, an important reaction for many physiological processes including respiration, fluid secretion and pH regulation. As such, hCA isoforms are prominent clinical targets for treating various diseases, such as glaucoma and epilepsy. hCA II is one of 12 catalytically active isoforms and, due to sequence conservation between them, substantial off-target binding to other isoforms occurs, reducing drug efficiency and causing side effects. Hence, there is a need to design effective hCA isoform-specific drugs. Over 400 X-ray crystal structures have been determined for hCA II, with around half of these in complex with inhibitors, yet despite the large amount of structural data available, key details regarding the H-atom positions of the protein and solvent and the charged state of the bound inhibitor were missing. The room-temperature neutron structure of hCA II in complex with AZM at 2.0 Å resolution revealed that the charged state of bound AZM was the anionic form, along with details of hydration, H bonding and hydro­phobic interactions between AZM and hCA II (Fisher *et al.*, 2012 ▸).

In this issue of **IUCrJ**, McKenna and co-workers (Aggarwal *et al.*, 2016 ▸) describe X-ray and neutron crystallography studies of hCA II in complex with a second sulfonamide inhibitor, methazolamide (MZM) – a methyl derivative of AZM that is preferred clinically for reasons such as greater stability, longer half life, lower dose and fewer side effects. They report the room-temperature neutron structure of hCA II in complex with MZM at 2.2 Å resolution, revealing the inhibitor is also bound in the anionic form, along with details of hydration, H bonding and hydro­phobic interactions between MZM and hCA II. They then compare the binding of the two inhibitors in the room-temperature neutron structures, and in particular show that more water molecules are displaced from the active site by MZM than AZM, relative to the neutron structure of unbound hCA II at 2.0 Å resolution (Fisher *et al.*, 2011 ▸). Since the overall binding affinity (*K*_i_) for both of the drugs against hCA II is similar (~10 n*M*), they discuss the balance between enthalpic and entropic contributions towards drug binding, and using molecular dynamics simulations suggest that in the case of MZM, hydro­phobic forces perhaps compensate for the loss of an extensive H-bonding network.

In recent years, a growing number of neutron structures have been deposited in the Protein Data Bank, including a number of other examples of enzyme-drug complexes, such as the recent structures of HIV-1 protease with the clinical inhibitors amprenavir (Weber *et al.*, 2013 ▸) and darunavir (Gerlits *et al.*, 2016 ▸), and the structure of farnesyl pyrophosphate synthase with the osteoporosis drug risedronate bound (Yokoyama *et al.*, 2015 ▸). Although the overall number of neutron structures is still relatively small, there are a growing number of examples for which neutron crystallography has provided the answers to questions that have remained elusive using other techniques.
